# Increased expression of HPV-E7 oncoprotein correlates with a reduced level of pRb proteins via high viral load in cervical cancer

**DOI:** 10.1038/s41598-023-42022-3

**Published:** 2023-09-12

**Authors:** Bilal Ahmad Mir, Arif Ahmad, Nighat Farooq, M. Vishnu Priya, A. H. Siddiqui, M. Asif, Rouquia Manzoor, Hassan Mubarak Ishqi, Suliman Y. Alomar, P. F. Rahaman

**Affiliations:** 1 Zoology Section, School of Sciences, Maulana Azad National Urdu University, Hyderabad, India; 2 Department of Radiation Oncology, MNJ Cancer Hospital, Hyderabad, India; 3 School of Medical Sciences, University of Hyderabad, Hyderabad, India; 4 Sher-i-Kashmir Institute of Medical Sciences, Soura Srinagar, J&K India; 5 Department of Surgery and Sylvester Comprehensive Cancer Center, Miller School of Medicine, University of Miami, Miami, FL USA; 6 Department of Zoology, King Saud University, 11451 Riyadh, Kingdom of Saudi Arabia

**Keywords:** Cancer prevention, Cancer screening, Gynaecological cancer, Tumour biomarkers, Tumour-suppressor proteins, Cancer, Diseases, Cancer, Urogenital diseases

## Abstract

Human Papillomavirus (HPV) is the most common cause of sexually transmitted diseases and causes a wide range of pathologies including cervical carcinoma. Integration of the HR-HPV DNA into the host genome plays a crucial role in cervical carcinoma. An alteration of the pRb pathways by the E7 proteins is one of the mechanisms that’s account for the transforming capacity of high-risk papillomavirus. For the proper understanding of the underline mechanism of the progression of the disease, the present study investigate the correlation of concentration of host pRb protein, viral E7 oncoprotein and viral load in early and advanced stages of cervical carcinoma. It was found that the viral load in early stages (stage I and II) was less (log_10_ transformed mean value 2.6 and 3.0) compared to advanced stages (stage III and IV) (Log_10_ transformed value 5.0 and 5.8) having high expression of HPV E7 onco-protein and reduced level of pRb protein, signifying the role of viral load and expression level of E7 oncoprotein in the progression of cervical cancer.

## Introduction

Cervical Cancer originates within the cells that line the cervix. It is primarily caused by the sequel to long-term, unresolved infection by certain genotypes of the human papillomavirus. The high-risk HPV-16 and HPV-18 subtype infections are the main types of HPV that are involved in the initiation and progression of the cervical carcinoma^1^. The HPV genome is non-enveloped, double-stranded DNA, approximately 8 kb in size, with eight open reading frames consisting of six early (E1, E2, E4, E5, E6, and E7) and two late (L1 and L2) genes that encode “early” (E) or “late” (L) proteins^2,3^. The E6 and E7 proteins have transforming properties that can convert normal cells into cancerous ones. Based on the High and Low-risk of causing cancer, more than 111 human papillomaviruses (HPV) types have been described. Among them, around 14 are classified as high risk including HPV-16, HPV -18, HPV-31, HPV-33, and HPV-45. The low risks include HPV-6 and HPV-11 which causes genital warts^4^.

HPV infection is essential but not sufficient for tumor induction^5^**.** Various studies indicate that HPV infection alone is not capable of transforming a normal epithelial cell to malignant one. The majority of HPV infections are asymptomatic and self-limiting hence could be naturally cleared in 70–90% of individuals with HPV infection. Hence genetic alterations in the genome of host cell seem to be required for the development and progression of cervical carcinoma.

HPV is probably the most common sexually transmitted disease agent. It is estimated that the worldwide age-standardized prevalence of current HPV infection is 10.5% in women; the prevalence varies about 20-fold between different regions, from 1.4% in Spain to 25.6% in Nigeria^6^. High-risk types, most notably HPV16 and HPV18, are the aetiological agent of cervical cancer. More than 35 HPV types are found in the genital tract, and HPV16 accounts for 50% to 60% of the cervical cancer cases in most countries including the United States and Europe, followed by HPV18 (10–12%) and HPVs 31 and45 (4–5% each)^7^. In a south Indian study, 87.8% high-risk HPVs positivity, with HPV-16 (66.7%), HPV-18 (19.4%), and other high-risk HPV types (1.7%), among HPV-positive cervical carcinoma patients was reported^8^.

HPV-16 integration into the host genome is a crucial step in the progression of cervical cancer which provides selective growth advantage compared to cells having HPV-16 viral particles as episomes^9–11^ This might be due to the interruption of the HPV-16 E2 open reading frame, leading to cellular immortalization by increasing the level of mRNAs of viral oncogenes E6 and E7^10,11^. The E2 gene is the most frequent site of disruption of viral DNA^12^. Host genome integration is believed to occur at some specific integration target sequences, the so-called genomic hotspots, where regions of microhomology (1-15bp, AT Rich) between viral and human genome sequences are found and are correlated with frequent fragile^13,14^ and transcriptionally active sites of the genome^15^.The E7 oncoproteins principally act through protein–protein interactions to change key pathways that regulate the progression of the cell cycle and cell proliferation by inactivating the retinoblastoma pocket proteins, suggesting that viral protein interactions with host cell proteins play a crucial role in regulating cell cycle progression in cervical carcinomas. The present study is designed to evaluate the risk of cervical cancer progression by quantifying E7 and pRb protein in the early and advanced clinical stages of cervical cancer and correlating it with the viral load. The analysis of clinically validated biomarkers such as HPV viral load, E7 proteins and cellular pRb profile in different stages of cervical cancer will help in detection of specific biomarkers for the screening of different stages of cervical cancer and a better understanding of the underline mechanism.

## Methodology

### Enrolment of patients

The cervical cancer patients recruited for the study were women who were attending Gynaecology OPD of MNJ cancer hospital Hyderabad. A total of 105 HPV-16 positive cervical cancer tissue samples and 30 HPV negative non-cancerous samples from patients who are suffering from some gynecological problems not related to cervical cancer were collected after taking approval from the institutional ethical committee of Maulana Azad National Urdu University Hyderabad and MNJ cancer hospital Hyderabad. All methods were performed in accordance with the relevant guidelines and regulations. Written informed consent was obtained from all the subjects and/or their legal guardians. The study subjects were aged 18–70.A portion of the collected biopsy tissue samples was formalin-fixed, paraffin-embedded, and used for histopathological examination and the other portion was stored in PBS vials at − 80 °C for viral load determination and western blot analysis. Stages of the cancerous cases were confirmed through histopathology using FIGO classification.

### Detection and quantification of high-risk HPV type 16 and 18 using real-time PCR

Genomic DNA was isolated from the histopathologically confirmed biopsy tissue samples of cervical cancer patients and controlled using salting out manual method. The HPV16/18 L1 amplicons were PCR-amplified from positive control genomic DNA using Taq polymerase and standard PCR conditions for cloning of HPV16 and 18 L1 amplicons. Both HPV16/18 L1 amplicons were cloned into plasmid vectors according to obtain the plasmids, pHPV16L1, and pHPV18L1, respectively. Purified plasmids were used to derive standard curves in the real-time PCR assay for the detection and quantification of HPV16 and 18 L1 amplicons.

### Sybr green real-time PCR

Real-time PCR reactions were performed using the Sybr Green master mix after standardizing the PCR conditions. For standard curves, real-time PCR was performed on purified plasmids, pHPV16 and pHPV18.

### Determination of relative amount of viral oncoprotein HPV- E7 and pRb protein level in HPV-infected cervical carcinoma tissue samples

Frozen Tissue biopsy samples were lysed using a protein lysis buffer. Total protein was measured by Bicinchoninic Acid (BCA) method using a BCA protein assay Kit (ratio 50:1), according to the manufacturer’s instructions (Thermo Scientific Pierce^TM^BCA Protein Assay Kit, Cat No 23225 and 23227) using bovine serum albumin as standard. Total proteins were separated by polyacrylamide gel electrophoresis using 10% resolving and 5% stacking gel and transferred to a nitrocellulose membrane.

The Western blotting technique was used to assess HPV16/18-E7 and host pRb protein levels. Anti-mouse pRb monoclonal antibody (ratio 1:1000 dilution: Thermo Fisher Scientific (Cat. No. LF-MA0173), and mouse anti-human papillomavirus 16/18 (E7) monoclonal antibody (1:100 dilution: Thermo Fisher Scientific (Cat. No. 28-0006 and Cat. No CF811359 respectively) were incubated with the Nitrocellulose membrane (NC) overnight at 4 °C. Antibodies against Glyceraldehyde 3-phosphate dehydrogenase (GAPDH) was used as an internal control for the target proteins and incubated under the same conditions (1:1000 dilutions: Thermo Fisher Scientific, Cat. No. AM4300). After incubation with the primary antibodies, the membrane was washed with PBST and the membrane was then incubated with goat anti-mouse secondary antibody (IgG H&L horseradish peroxidase: 1:5000 dilution, Elabsciences, China cat No E-AB-1001) for one hour. The membranes were washed with PBST and the protein bands on the membrane were detected using enhanced chemiluminescence substrate (Elabscience, China Cat NoE-BC-R347) for one minute according to the manufacturer’s instructions and the bands were visualised in ChemiDoc (Bio-Red). The bands on the membrane were analyzed by densitometric analysis using IMAGE J Software. The NC membrane was cut horizontally prior to hybridization with primary antibodies according to the molecular weight of 3 different (pRb, HPV-E7, GAPDH) proteins to quantify from a single blot, and incubated separately with specific primary and its corresponding secondary antibodies for images development.

### Ethical approval

The present study was undertaken after approval from the institutional ethical committee of Maulana Azad National Urdu University Hyderabad and MNJ cancer hospital Hyderabad. Written informed consent was obtained from all the subjects and/or their legal guardians.

## Results

One hundred and five cervical cancer patients with at least one positive marker of cervical abnormalities were recruited for the study. All patients were south Indian aged 30–90. According to histological findings, 07 patients were diagnosed with stage I, 53 patients were diagnosed with stage II, 30 patients were diagnosed with stage III and 15 patients were diagnosed with stage IV. In cervical carcinoma, all samples are HPV-16 positive including 8 samples co-infected with HPV-18.

### HPV16 viral load increases with the grade of the lesion

**Figure 1:**
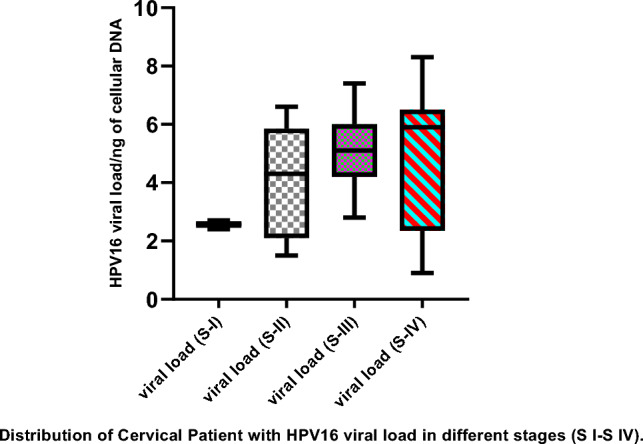
Box and Whisker plot showing Distribution of HPV16 viral load in different stages of cervical cancer. Advanced Stages III and IV show significantly higher viral load compared to early stages I and II of cervical carcinoma. Viral load values are converted into log_10_ values. S-I to S-IV are FIGO stages of cervical cancer.

The number of HPV16 viral genomes (viral load) per ng of cellular DNA (log_10_ value) was evaluated in 105 cervical cancer patients in different stages. We observed that the median HPV16 viral load increases with the lesion grade. In Stage I, viral load ranged from 240 copies/µg of DNA (log_10_ value 2.4) to 460 copies/µg of DNA (log_10_ value 2.7) with a median viral load of log_10_ value 2.6. In stage II, viral load ranged from 122 copies/µg of DNA (log_10_ value 2.1) to6624665 copies/µg of DNA) (log_10_ value 6.8) with a median viral load of log_10_ value 4.4.In stage III, viral load ranged from 659 copies/µg of DNA (log_10_ value 2.8) to 26,213,401 copies/µg of DNA) (log_10_ value 7.4) with a median viral load of 4.9 and in Stage IV, viral load ranged from 788 copies/µg of DNA (log_10_ value 2.9) to198908450 copies/µg of DNA (log_10_ value 8.3) with a median viral load of 5.2 were detected. Figure 1 represents the distribution of HPV16 viral load in different stages of cervical cancer. Statistical analysis revealed significant differences in viral loads between early stages (stage I and II) and advanced stages (stage III and IV) of cervical cancer (*p value* < 0.001) as shown in Table 1. The highest HPV16 loads (median load of 43,958,767,572 copies (per µl of DNA) were observed for biopsy samples from cervical cancers stage IV. These values are significantly higher than those observed in the early stages (stages I and II) of cervical cancer.

**Table 1:** Tukey multiple comparisons test to check the level of significance of viral load in different stages of cervical cancer.

Stages	Mean Difference	95% CI of diff	Below threshold?	Summary	*p*-value (Adjusted)
I versus II	− 1.827	− 2.8 to − 0.82	Yes	***	< 0.001
I versus III	− 2.361	− 3.4 to − 1.2	Yes	***	< 0.001
I versus IV	− 2.574	− 3.6 to − 1.4	Yes	***	< 0.001
II versus III	− 0.53	− 1.4 to 0.35	No	ns	0.405
II versus IV	− 1.74	− 1.6 to 0.14	Yes	**	0.003
III versus IV	− 0.21	− 1.2 to 0.79	No	ns	0.946

Tukey's multiple comparisons test was used to compare HPV16 viral load among different stages of cervical cancer tissue samples, A significant difference in viral load was found between stage I and stage III (mean difference − 1.82, *p*-value < 0.001), stage I and stage II (mean difference − 2.36, *p*-value < 0.001), stage II versus Stage IV (mean difference − 1.7, *p*-value 0.003) and stage I and stage IV (mean difference − 2.57, *p*-value < 0.001).

**Figure 2:**
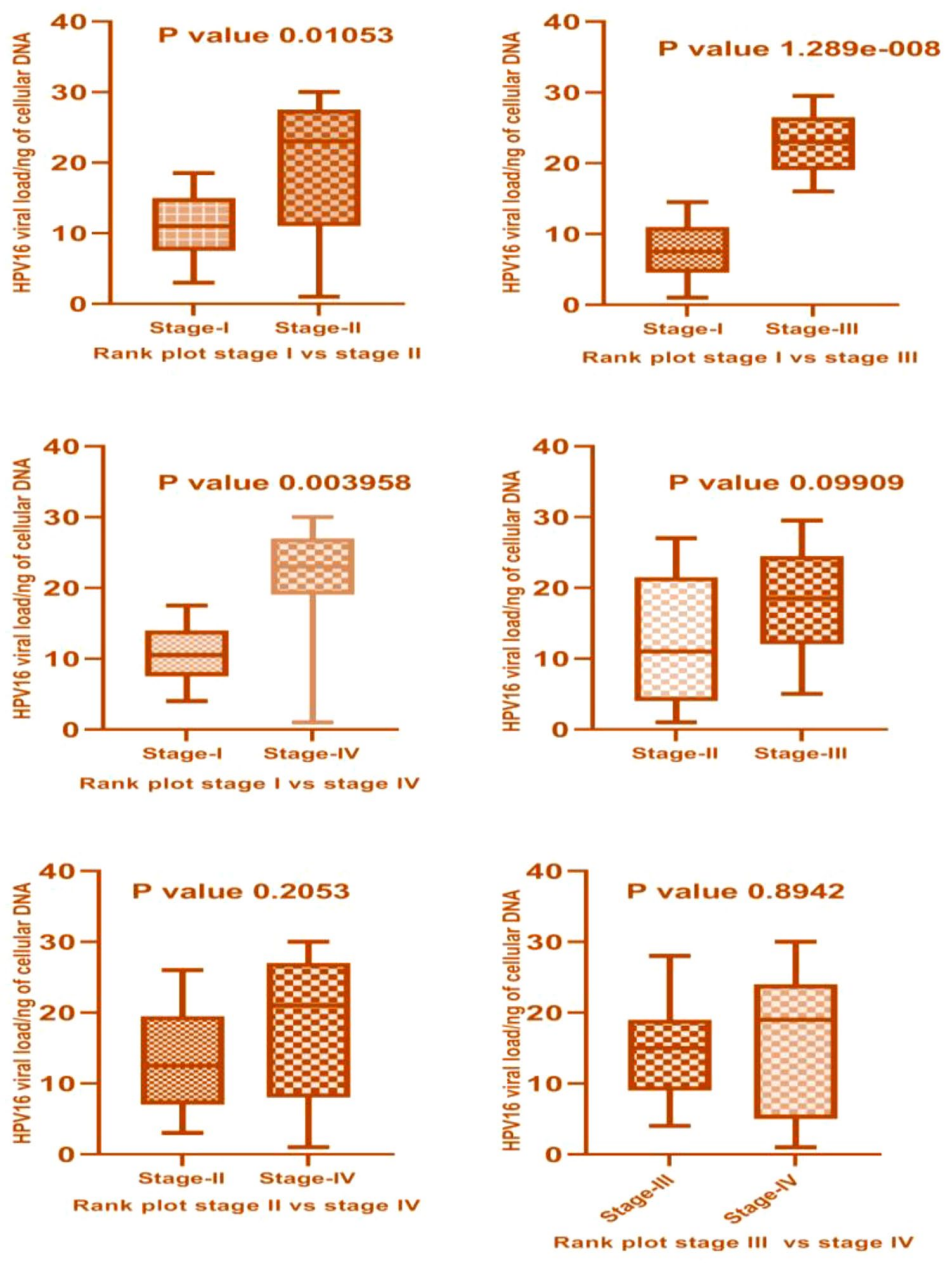
Mann- Whitney U test for the comparative analysis of viral load in different FIGO stages of cervical cancer. Significantly high viral load is observed in advanced stages (III and IV) compared to early stages (I and II) of cervical cancer and also within the early and advanced stages of cervical cancer.

Mann–Whitney U-Test, a nonparametric form of T-test was used to check the difference in viral load in different stages of cervical cancer. HPV16 viral load in stage II was high and statistically significant in comparison to stage I cervical cancer cases (*p*-value 0.010). Also, a high and significant viral load was found in stage III versus stage I (*p*-value < 0.001) and stage IV *versus* stage I cervical cancer cases (*p*-value 0.003). No significant difference in HPV16 viral load was found between stage III and IV (*p*-value 0.894) as shown in Fig. 2.

### Expression analysis of pRb tumor suppressor protein in different stages of cervical cancer

The retinoblastoma protein (pRb) is a nuclear phosphoprotein that plays an essential role in regulating the cell cycle. HPV E7 protein has been proposed to interact with host pRb tumor suppressor protein, thus abrogating its function. The expression levels of pRb in different stages of cervical cancer cases were measured relative to normal non-cancerous tissue samples. In stage I, 5/7 (71.4%) samples showed significantly high expression of pRb (*P* value < 0.0090) while two samples showed normal expression. In stage II, 43/53 (81.03%) samples had no significant difference in pRb expression when compared with stage I (*P* value < 0.0917), but a significant difference was found when compared to normal non-cancerous tissue samples (*P* value < 0.004) while ten samples showed normal expression. In higher stages, the expression of pRb was significantly less in 24/30 (80%) cases in stage III (*P* value 0.0001) and 13/15 (86%) in stage IV (*P* value < 0.001) when compared to normal non-cancerous cases. Also, a less statistically significant difference was found in the expression of pRb of stage III (P value 0.030) and stage IV cervical cancer cases (*P* value < 0.0001) compared to normal non-cancerous cases. Rest 8 samples of higher stages shows normal expression.

The pairwise Tukey multiple comparisons test that was used to check the mean difference and level of significance of retinoblastoma tumor suppressor protein (pRb) between normal control and different stages of cervical cancer cases (Table 2). The highest mean difference was found between stage I versus stage IV (mean difference 0.3459), *p*-value (< 0.0001), stage I versus III (mean difference 0.2519), *p*-value (< 0.0001), and between normal control versus stage IV (mean difference 0.1952), *p*-value (< 0.0001) and the lowest mean value were found between normal control versus stage II (mean difference − 0.094), *p*-value (0.004), Normal control versus stage I (mean difference − 0.1507), *p*-value (0.0090) and between stage III versus stage IV (mean difference 0.094), *p*-value (0.097).

**Table 2:** Tukey's multiple comparisons test between the expression of pRb protein in Normal Control (NC) versus different stages of cervical carcinoma.

pRb in NC and Stages	Mean difference	95% CI of difference	Significant?	Summary	*p* Value (Adjusted)
NC-versus I	− 0.15	− 0.27 to − 0.02	Yes	**	0.009
NC-versus II	− 0.08	− 0.17 to 0.008	Yes	**	0.004
NC-versus III	0.10	0.006 to 0.19	Yes	*	0.03
NC-versus IV	0.19	0.10 to 0.29	Yes	****	< 0.0001
I versus II	0.06	− 0.063 to 0.19	No	ns	0.61
I versus III	0.25	0.12 to 0.38	Yes	****	< 0.0001
I versus IV	0.34	0.21 to 0.47	Yes	****	< 0.0001
II versus III	0.18	0.083 to 0.28	Yes	****	< 0.0001
II versus IV	0.27	0.17 to 0.38	Yes	****	< 0.0001
III versus IV	0.09	− 0.010 to 0.19	No	ns	0.0975

**Figure 3:**
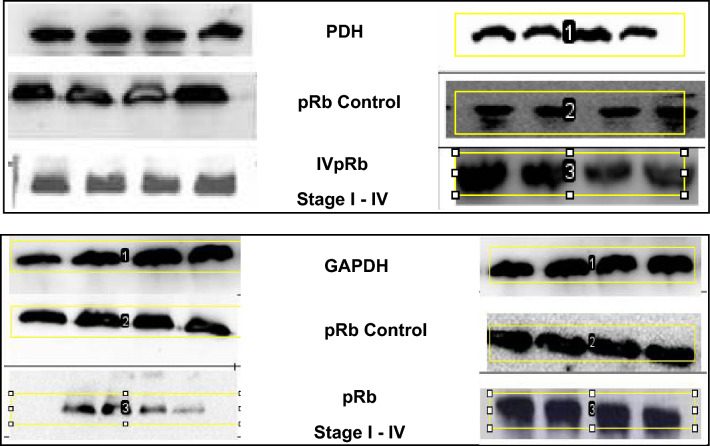
Western blot images of the expression level of pRb protein normalized with GAPDH protein. Each group has four control samples (2nd Rows) and four cancerous samples of stages I to IV (3rd rows). Densitometric analysis of the bands showed higher expression of pRb in early stages (I and II) compared to advanced stages (III and IV) of cervical cancer. (The images are cropped. For complete images please see the supplementary data).

**Figure 4:**
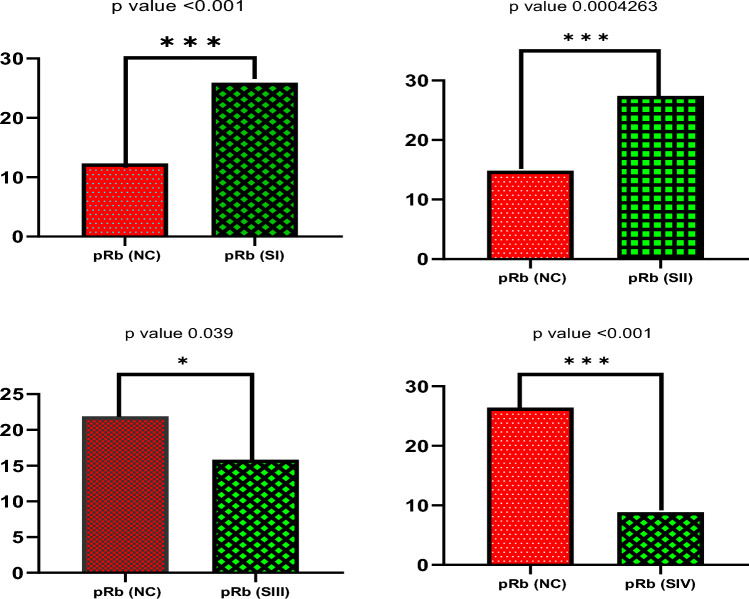
Mann–Whitney rank correlation test of expression of pRb tumor suppressor proteins in normal control and different stages of cervical cancer cases. Significant differences in the expression of pRb in normal control samples were found compared to the pRb expression in different stages of cervical cancer. In the early stages, expression of pRb protein is higher compared to compare to normal control samples. However, in advanced stages expression of pRb protein is lower compared to normal control samples.

Based on Mann–Whitney U-Test, it was observed that the expression of pRb tumor suppressor protein varies significantly within different stages of cervical cancer compared to normal non-cancerous tissue samples. Stage I and II show a high and significant difference in pRb expression compared to normal non-cancerous tissue samples (*p* value < 0.001), however in late stage III and stage IV, the expression of pRb is less and statistically significant compared to normal non-cancerous tissue samples (*p*-value 0.039 and < 0.0001 respectively) as shown in Figs. 3 and 4 representative images below.

### Expression analysis of HPV E7 relative to pRb tumor suppressor proteins in different stages of HPV-infected cervical cancer tissue samples

The Retinoblastoma protein is a well-known molecular target of the E7 oncoprotein of high-risk papillomavirus. E7 has been shown to modulate the pRb function in two ways. Primarily it dislodge pRb from its complexes with cellular regulatory proteins like transcription factor E2F, resulting in the activation of genes important for cell cycle regulation, cell cycle progression, and DNA replication. Secondarily, E7 induce pRb degradation which is expected to have a similar impact on cell cycle regulation. Both mechanisms were identified and investigated extensively in cultured cell lines, but little is known about the molecular pathway through which E7 overrides pRb function in human malignancies.

**Figure 5:**
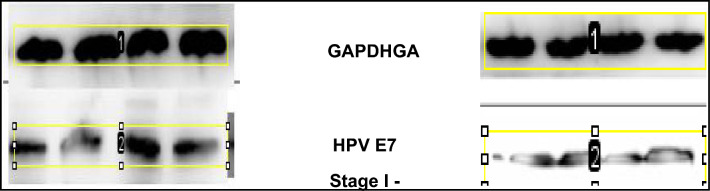
Western blot images of the expression level of HPV-E7 protein in four cervical cancer tissue samples of stage I to IV normalized with GAPDH protein. Densitometric analysis of the bands showed lower expression of HPV-E7 protein in early stages (I and II) compared to advanced stages (III and IV) of cervical cancer. (The images are cropped. For complete images please see the supplementary data).

**Figure 6:**
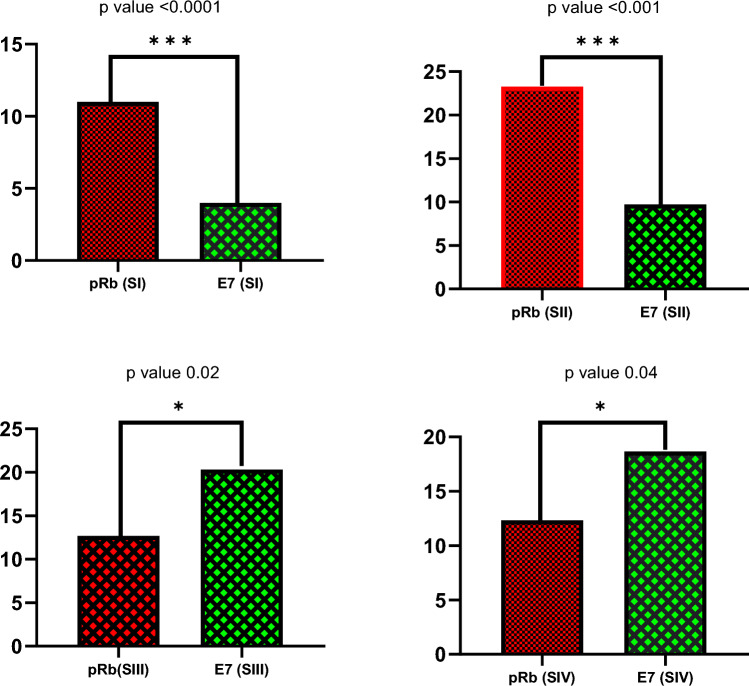
Mann–Whitney rank correlation test of expression of HPV E7 and pRb tumor suppressor proteins in different stages of cervical cancer cases. In the top panel (Early stage I and II) low expression of HPV-E7 protein compared to pRb protein was detected (*p*-value 0.00058 and < 0.001). However, in advanced stages III and IV, the expression of HPV-E7 protein is significantly higher compared to pRb protein (*p*-value 0.02 and 0.048 respectively).

Using Mann–Whitney U-Test it was identified that the expression of HPV E7 protein with respect to pRb tumor suppressor protein varies significantly within different stages of cervical cancer. Stage I and II show statistically significant less expression of HPV-E7 compared to expression of pRb tumor suppressor protein (*p* value 0.00058 and < 0.001) with reference to GAPDH protein. However, in advanced stage III and stage IV, the expression of E7 is statistically significant high compared to pRb expression (*p*-value 0.02 and 0.048 respectively) with reference to GAPDH protein. (Fig. 5). These findings suggest that pRb is degraded in the advanced stages of cervical cancer, by increased proteolytic degradation triggered by E7; hence the relative abundance of E7 and pRb is inversely correlated (Fig. 6).

To check the association of HPV E7 with pRb protein expression, Pearson’s r Correlation matrix was used to measure the strength of the linear relationship between pRb and HPV E7 expression in a different stage of cervical cancer. Inverse correlation (− 0.487) was found between stage I pRb and stage I HPV E7 (− 0.053) and stage II pRb and stage II HPV E7. Also, expression of HPV E7 between stage I and III (− 0.18) and stage I and IV (-0.15) were found significantly negatively correlated (Table 3).

**Table 3:** Pearson’s r Correlation test of HPV E7 and pRb tumor suppressor protein between early stage (stage I and II) and advanced stage (stage III and stage IV) of cervical cancer.

	pRb I	E7 I	pRb II	E7 II	pRb III	E7 III	pRb IV	E7 IV
pRb,I	1	− 0.06	0.45	− 0.53	− 0.58	0.53	0.25	− 0.24
E7, I	− 0.06	1	− 0.04	− 0.01	− 0.16	− 0.18	0.30	− 0.15
pRb, II	0.45	− 0.04	1	− 0.113	− 0.34	0.26	0.39	− 0.14
E7, II	− 0.53	− 0.012	− 0.11	1	0.25	− 0.023	− 0.007	0.41
pRb, III	− 0.58	− 0.16	− 0.34	0.25	1	− 0.34	− 0.68	0.16
E7, III	0.53	− 0.18	0.26	− 0.023	− 0.34	1	0.13	0.27
pRb, IV	0.25	0.30	0.39	− 0.007	− 0.68	0.13	1	0.005
E7, IV	-0.24	− 0.15	− 0.14	0.41	0.16	0.27	0.005	1

### Correlation of HPV E7 viral proteins and host pRb tumor suppressor proteins with viral load among different stages of cervical cancer tissue samples

**Figure 7:**
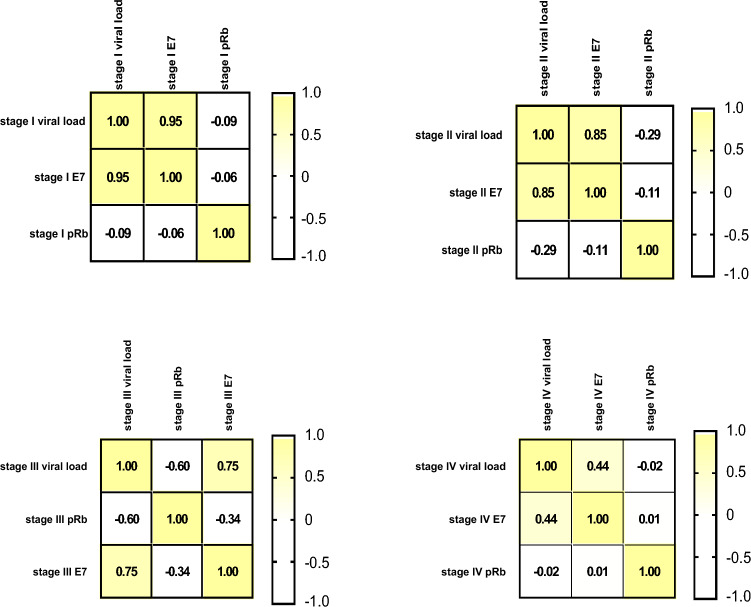
Correlation matrix of HPV16 viral load, HPV-E7 viral protein, and host pRb protein in different stages of cervical cancer. The figure showed a positive correlation between the expression of HPV-E7 viral protein with HPV viral load and an inverse relationship with pRb protein expression in early stages I and II to advanced stages III andIV of cervical cancer.

There is a positive correlation between viral load and expression of HPV E7 oncoproteins in early and advanced stages of cervical cancer and elevated expression of HPV E7 correlates with reduced pRb levels in different stages of cervical cancer. Figure 7 illustrates stage wise correlation of viral load with HPV E7 oncoproteins and host pRb tumor suppressor proteins in the early stage (stage I and stage II) and late stage (stage III and stage IV) of cervical cancer**.**

## Discussion

Cervical cancer is one of the most common types of cancer in females. Human Papilloma Virus (HPV) is one the prime cause of cervical cancer. High-risk HPV-16/18 is involved in the multistep pathogenesis of cervical carcinoma. Along with High-risk HPV-16/18, the progression of cervical cancer can be influenced by the host immune system and other carcinogens^16^. Pre-neoplasia may regress or progress to an invasive cervical carcinoma after a long period of latency. The HPV genome encodes oncoproteins that induce cellular proliferation and inhibit apoptosis^17^.

The etiopathologic association of high-risk HPV infection with squamous cell carcinoma of the uterine cervix has been well documented in several studies^18–20^. In our study, we included only HPV-positive cases as the main aim of our study was the determination of viral load and its association with the progression of the disease. The number of HPV16viral genomes (viral load) per ng of cellular DNA (log_10_ transformed value) was evaluated in 105 cervical cancer patients in different stages. The association between the viral load and the different phases of disease is still debated. We found a higher HPV16 viral load associated with advance stages of cervical cancer. The HPV-16 viral load increased with the severity of the disease, with significant differences between early and late-stage carcinoma. Most studies have observed that higher HPV viral loads are associated with augmented abnormal cytological or histological Diagnosis^21–28^. Although some studies did not observe a positive association between the viral load and the severity of the disease^29,30^. The differences in results may be due to different methods used to measure and analyze viral loads and also the range of viral load in different patients is enormous. The studies with the most comparable reporting to ours were that of^31–34^ who found increased HPV16 viral load with the increased severity of the disease.

The known mechanism of loss of normal Rb function is either due to mutation in Rb gene or binding of HPV-E7 protein to pRb-E2F complex which removes cell cycle restriction. HPV-16 E7 protein interacts with and functionally inactivates the tumor suppressor protein pRb and destabilizes the Rb-E2F complex resulting in the release of an active E2F transcription factor. The free E2F stimulates the cell to enter into the S phase of the cycle. It is reported that the E7 protein is found predominantly in the nucleus and in small amounts in the cytoplasm of the cervical cancer cell line CaSki and cervical carcinoma cells, suggesting an important role of E7 in the development of cervical carcinoma^35,36^. The functional state of pRb depends on the state of its phosphorylation, which occurs in a cell cycle-dependent manner. The hypophosphorylated form of pRb functions as a cell cycle regulator. It is also known that several adenoviruses and other DNA viruses proteins including the SV40 large tumor antigen and HPV E7 protein bind to the hypophosphorylated form of pRb^37–41^. The binding of viral oncoproteins to hypophosphorylated form pRb is thought to functionally inactivate its tumor-suppressor activity. The expression of pRb has not been intensively studied in human cervical cancers along with the HPV status.

Several studies in different cancerous and normal tissues employing both protein and mRNA analysis, have indicated that the Rb gene is ubiquitously expressed^42–48^. However, such studies were not able to distinguish between different cell types within the tissues analyzed. Some immunohistochemical studies on pRb in various human cancer and normal tissues including the uterine cervix shows that pRb is expressed in mature and differentiated cells^49,50^. According to a study conducted by^51^, pRb expression is relatively less in CIN III and Squamous Cell Carcinoma compared to the normal squamous epithelium and squamous metaplasia.

In the present study,an inverse relationship between pRb and HPV E7 protein in the early and advanced stages of cervical carcinoma was detected (Fig. 7). pRb was overexpressed in 79.4% of early-stage cervical cancer samples (Stage-I and II). Significantly high expression of pRb was found in early-stage cervical tissue samples compared to pRb expression of the advanced stage of cervical cancer (*p*-value < 0.001).Also, pRb expression of the early stage of cervical cancer tissue samples was found high than the pRb expression of normal noncancerous tissue samples (adjusted *p*-value 0.005). In contrast to previous reports (normal pRb expression in cervical carcinomas^52,53^, we found expression of pRb is lower in advanced stages of invasive cervical carcinoma compared to early stages. This suggested that Rb protein inactivation/degradation could be involved in advanced stages of cervical carcinogenesis and is supposed to be related to pRb complex formation with high-risk HPV E7 oncoprotein resulting in its degradation. Similar results were also reported in some of the previous studies^54,55^.

In our study, an inverse relation between pRb and E7 protein both in the early and advanced stages of cervical cancer was observed. In the early stage, pRb is overexpressed and E7 expression is less (r-value − 0.18, *P*-value 0.04) in contrast to advanced stages (stage III and IV) of cervical cancer where pRb expression is very less but the expression level of HPV E7 is found significantly high then pRb (adjusted *p*-value 0.03). Low expression of E7 protein in the early stage of cervical cancer possibly results from the host's factor preventing the E7 gene expression at transcriptional, translational, and/or posttranslational levels.

The viral load may also be another important factor in determining the expression levels of E7 protein in different stages of cervical carcinoma as a high viral load was found and associated with a high grade of the cervical lesion. The advanced stages are associated with a high viral load compared to the early stages. The variable levels of E7 oncoprotein expression in different cells might contribute to the heterogeneity. Changes in pRb expression in individual cells could be observed as they undergo G0/middle G1 phases of the cycle^56,57^. In cells having high HPV16 viral infection (different E7 oncoprotein concentrations), the pRb detection could be very low to undetectable as we found in advanced cervical carcinoma samples. Other types of human cancers are also associated with altered pRb expression patterns^58^. Invasive cancers of early clinical stages express high pRb levels^59^. However, late clinical stages of invasive cancers express low to the undetectable level of pRb^60,61^.

## Conclusion

In conclusion, the HPV viral load tends to be higher in women with high-grade cancer diagnoses, especially for HPV16. The HPV16 viral load increased with the severity of the disease, with significant differences between early and late-stage carcinoma. There is an inverse relationship between the expression of HPV 16 E7 and pRb protein in early (stage I and II) and advanced stages (stage III and IV) of cervical cancer. There is a positive correlation between viral load and expression of HPV E7 oncoproteins in early and advanced stages of cervical cancer and a high level of HPV E7 expression correlates with reduced pRb levels in different stages of cervical cancer. Therefore, a combination of HPV viral load and expression status of its E7 proteins along with host pRb expression may be used as a potential diagnostic biomarker for screening and characterization of the early stages of cervical carcinoma and management of the disease.

### Supplementary Information

Supplementary Figures.

## Data Availability

Raw data is available with the corresponding author on reasonable request. We are not making it publicly available as Dataset Generated or analysed during the current study are complicated and intricate calculations are involved.
