# Supplementary material for: Increased expression of HPV-E7 oncoprotein correlates with a reduced level of pRb proteins via high viral load in cervical cancer
Source: Sci Rep. 2023 Sep 12;13:15075. doi: 10.1038/s41598-023-42022-3 (PMC10497568; doi:10.1038/s41598-023-42022-3)
Supplement: Supplementary file 1 — Supplementary Figures. [file 41598_2023_42022_MOESM1_ESM.docx]

**ORIGINAL IMAGES OF FIGURE 3**


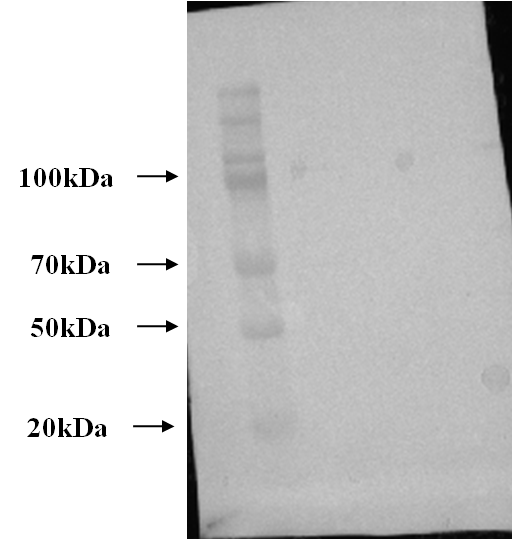


**1. Protein Marker:**

**2. Original Representative images of pRb (Normal Control) (110 Kd). Each band represents different sample for pRb expression.**


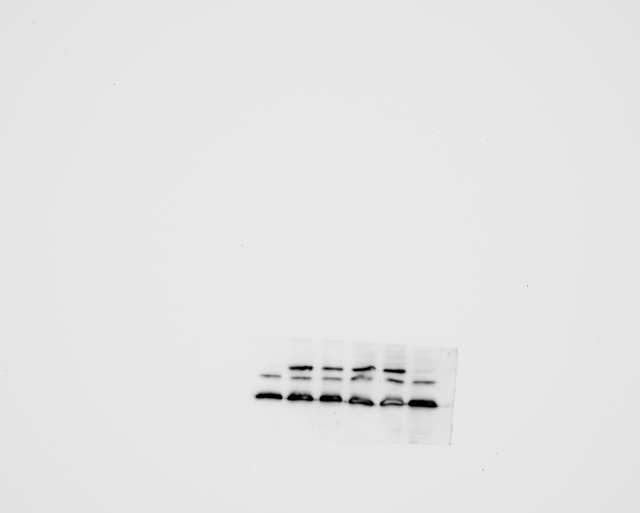

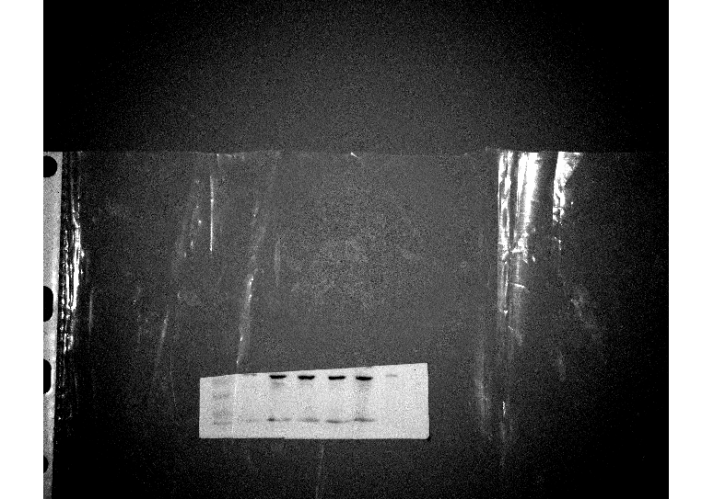


**pRb (control)**

**pRb (control)**


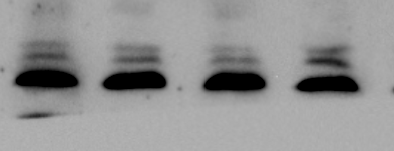

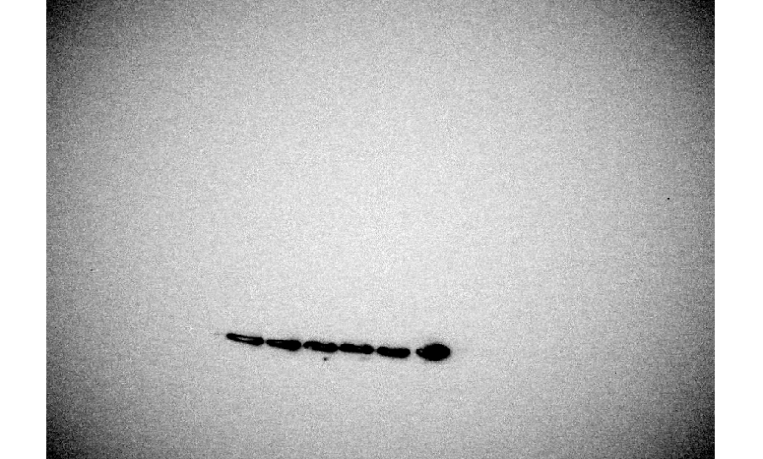


**pRb (control)**

**pRb (control)**


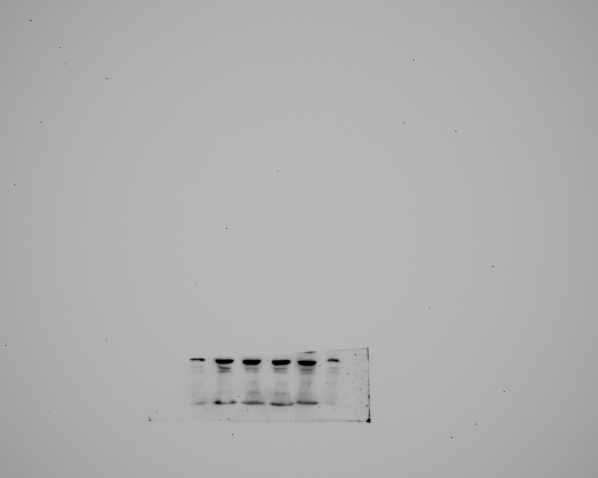


**pRb (control)**

3. **pRb Cervical Cancer (110 kd) Left to Right: Stages I-IV**

**
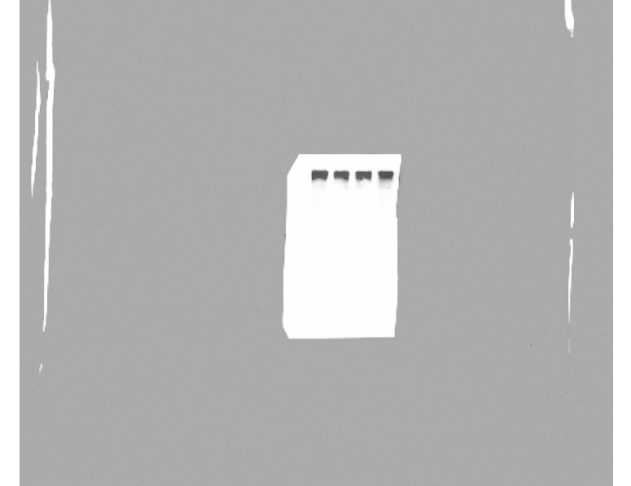

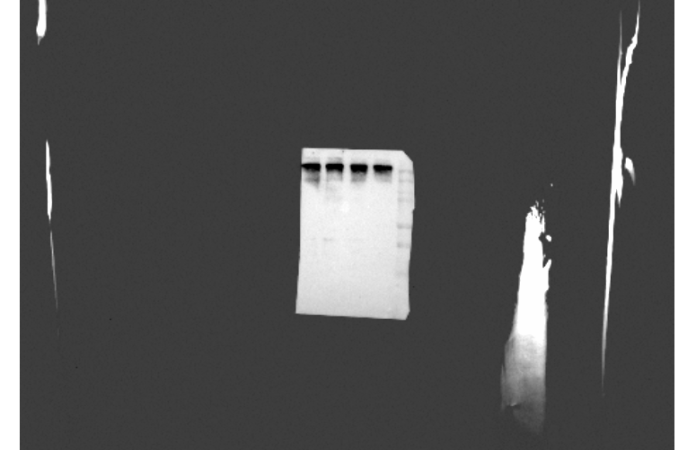
**

**I II III IV**

**I II III IV**

**pRb (SI-S IV)**

**pRb (SI-S IV)**

**pRb (SI-S IV)**

**I II III IV**

**I II III IV**

**
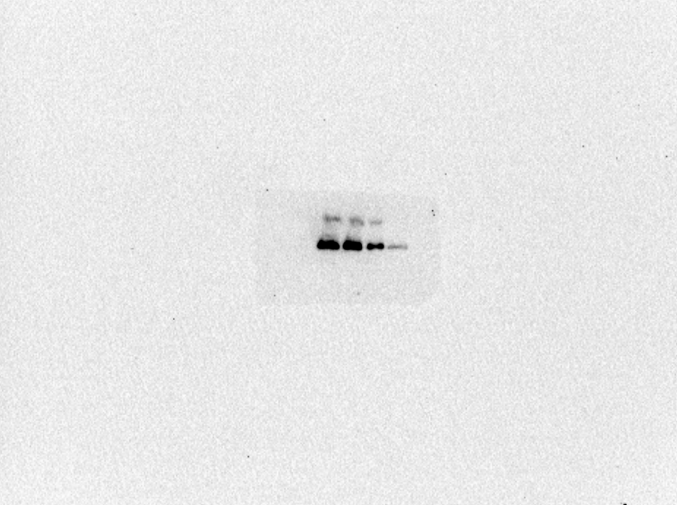

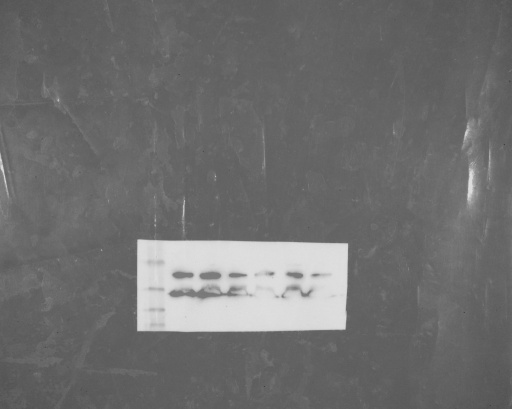
**

**pRb (SI-S IV)**

**4. GAPDH (40 kd) internal Control:**

**
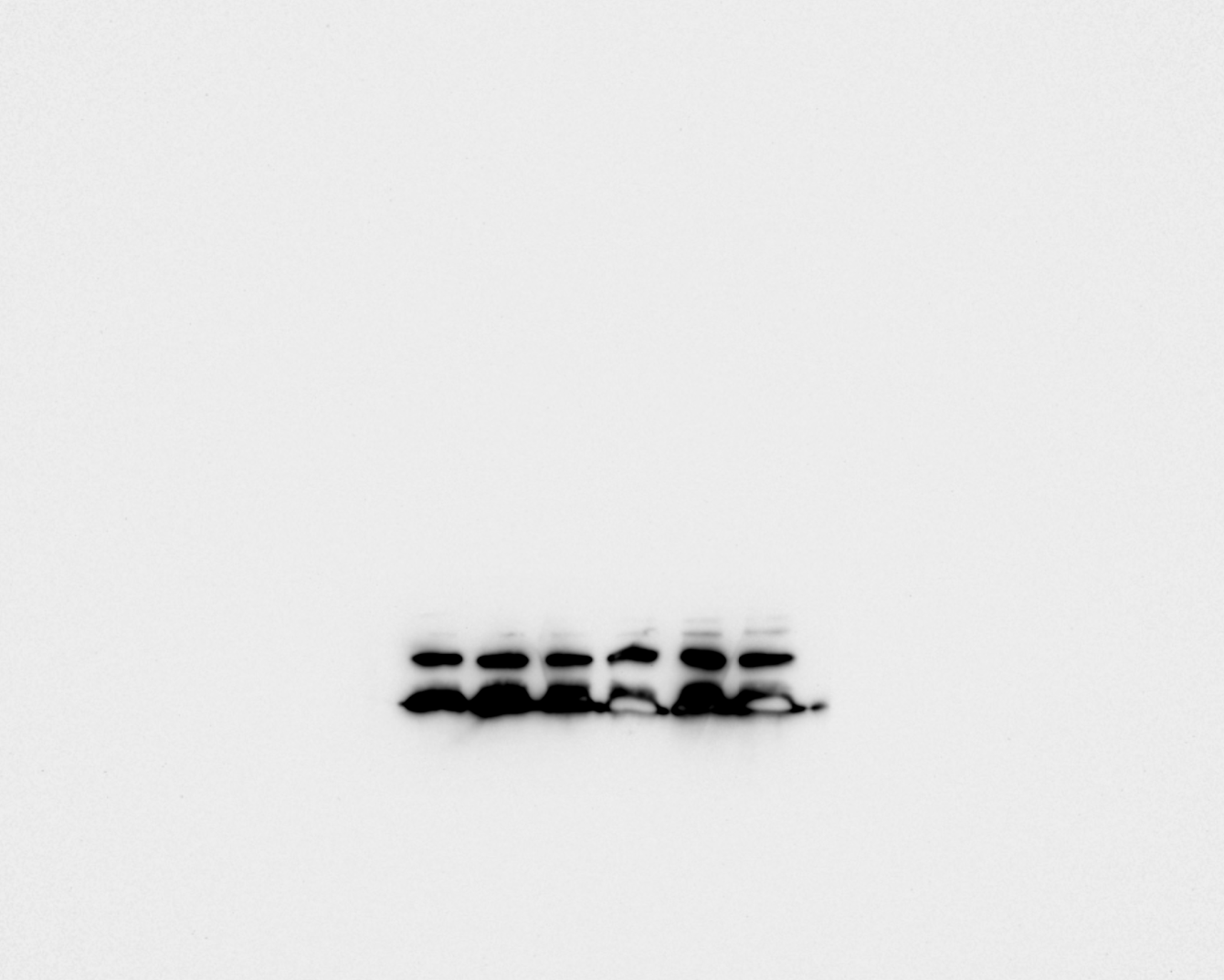

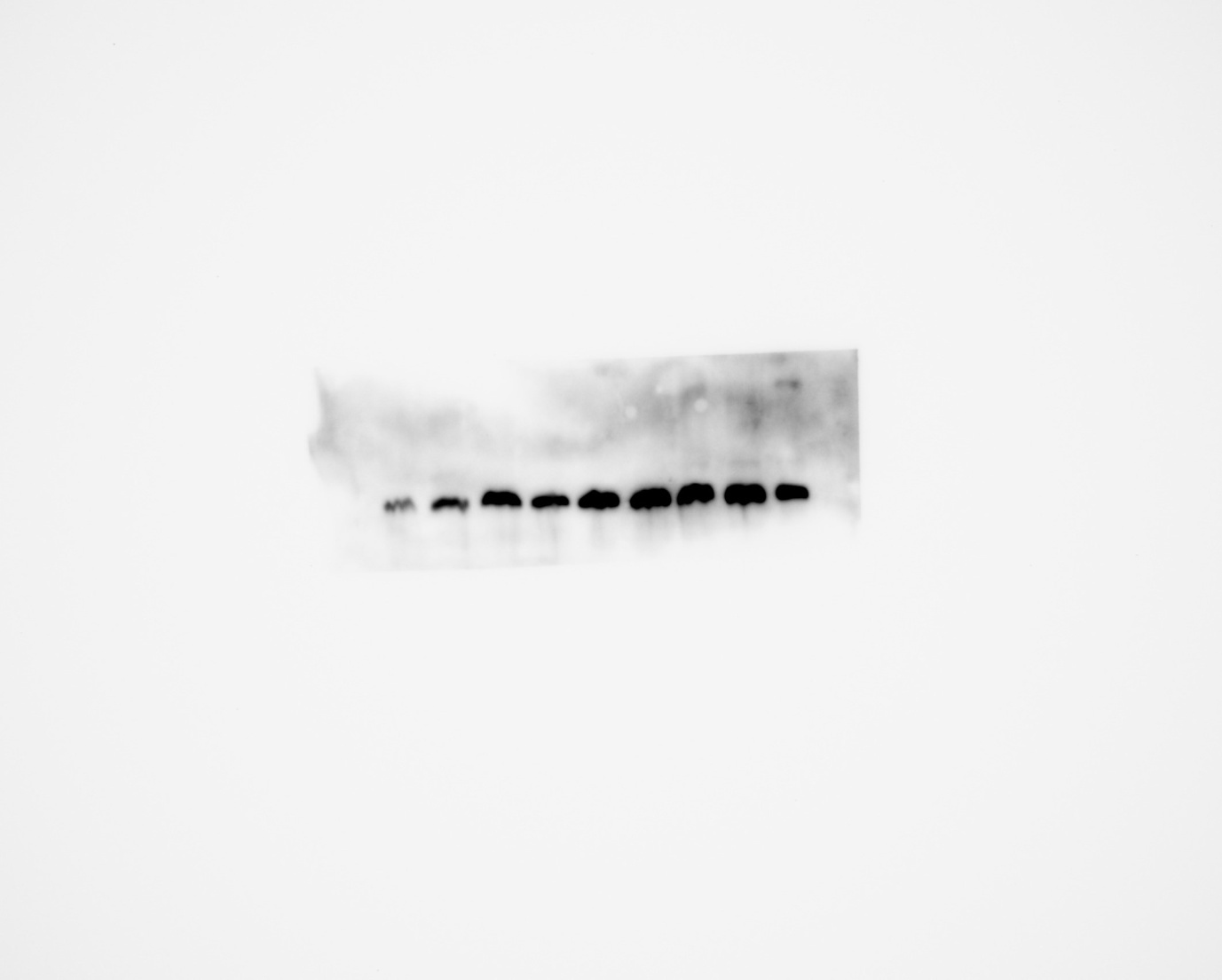
**

**
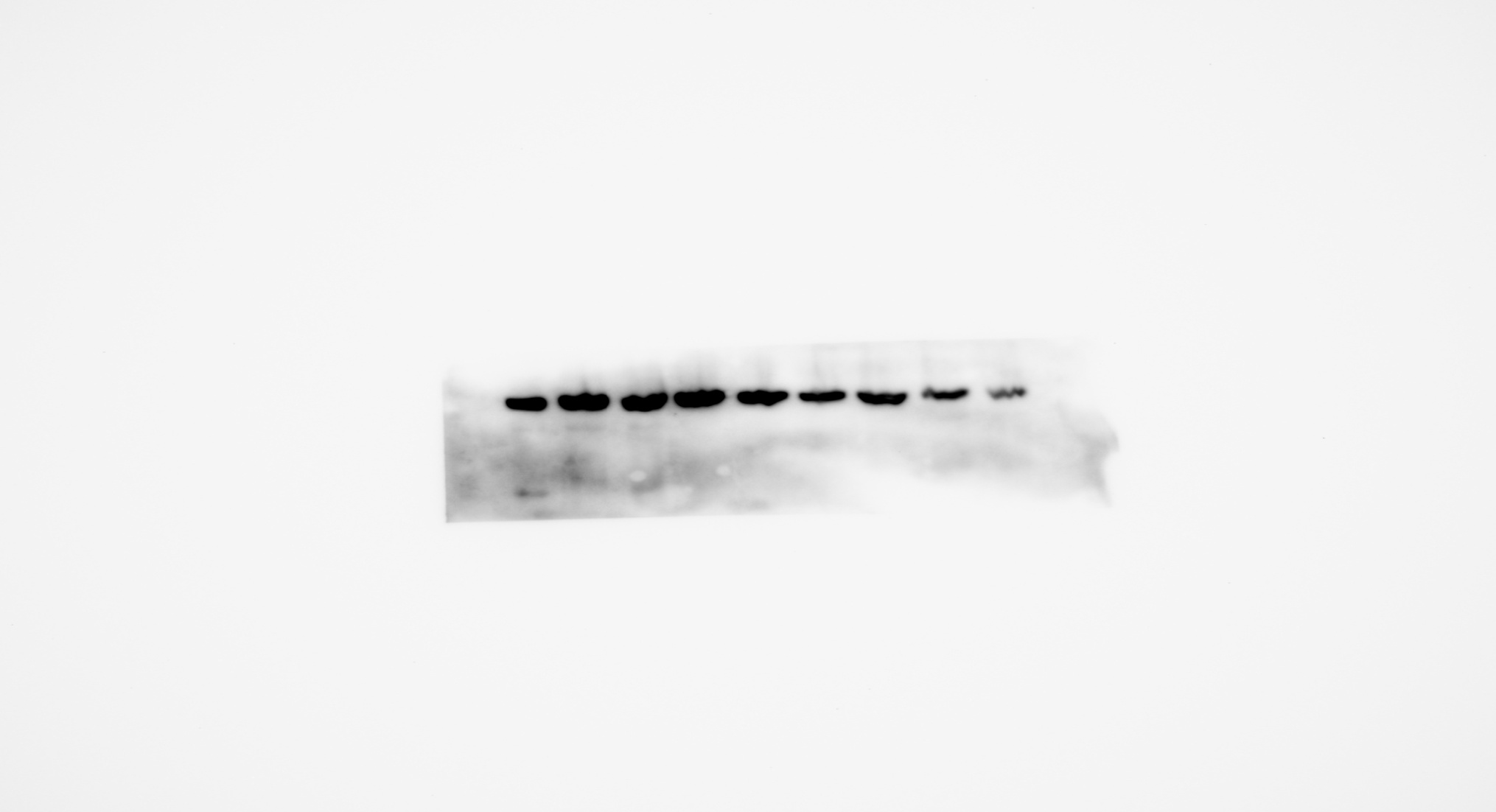
**

**42kD**

**GAPDH**

**42kD**

**GAPDH**

**GAPDH**

**ORIGINAL IMAGES OF FIGURE 5**

**
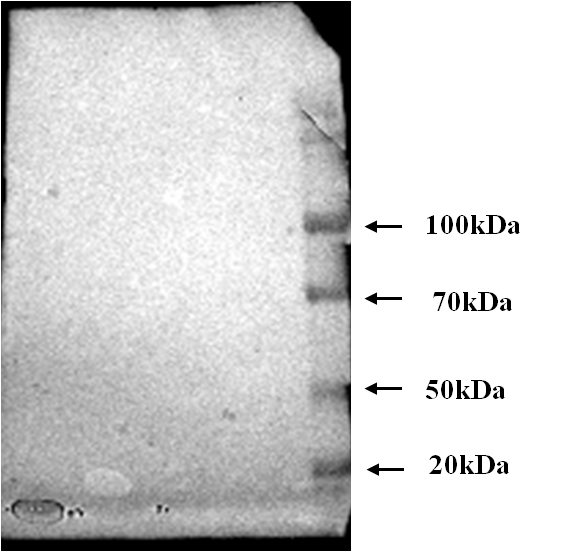
5. Protein Marker:**

**6. Representative western blotting images of HPV-E7 (Stages I-IV ) in different cervical cancer tissue samples**

**
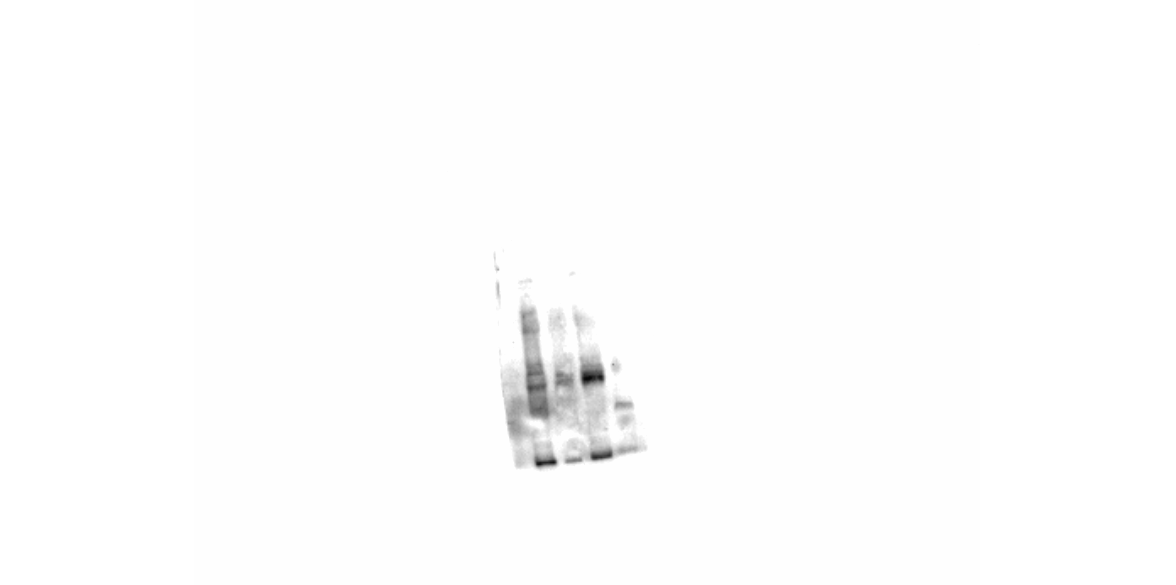
**

**I II III IV**

**HPV E7 (SI-S IV)**

**HPV E7 (SI-S IV)**

**I II III IV**

**
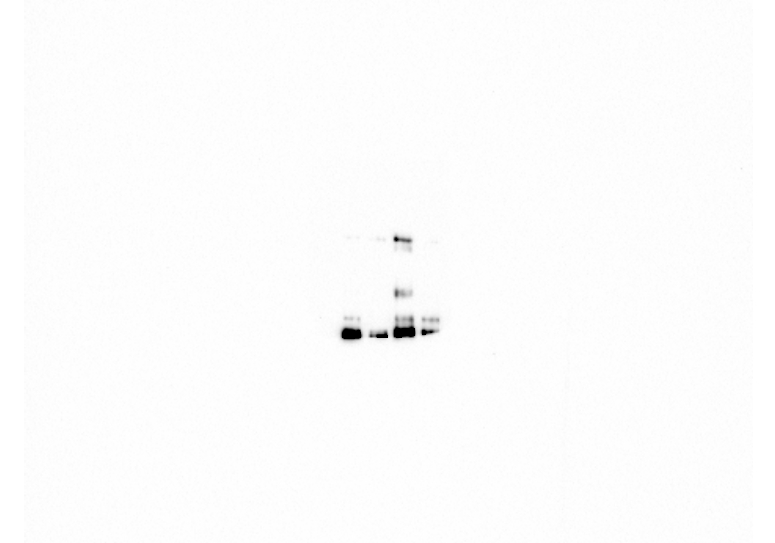

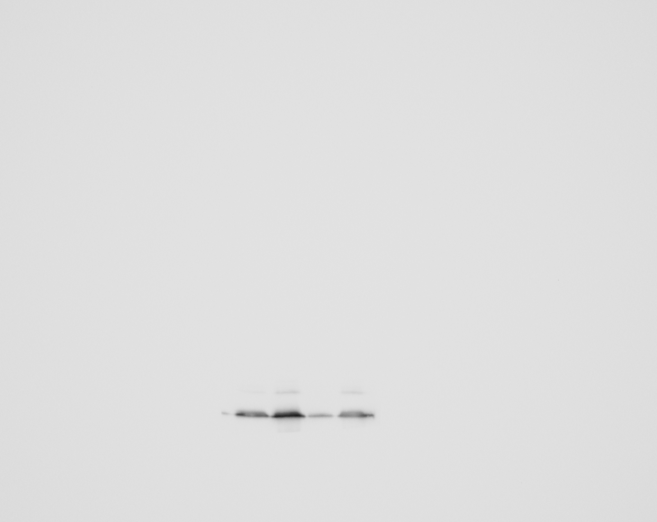

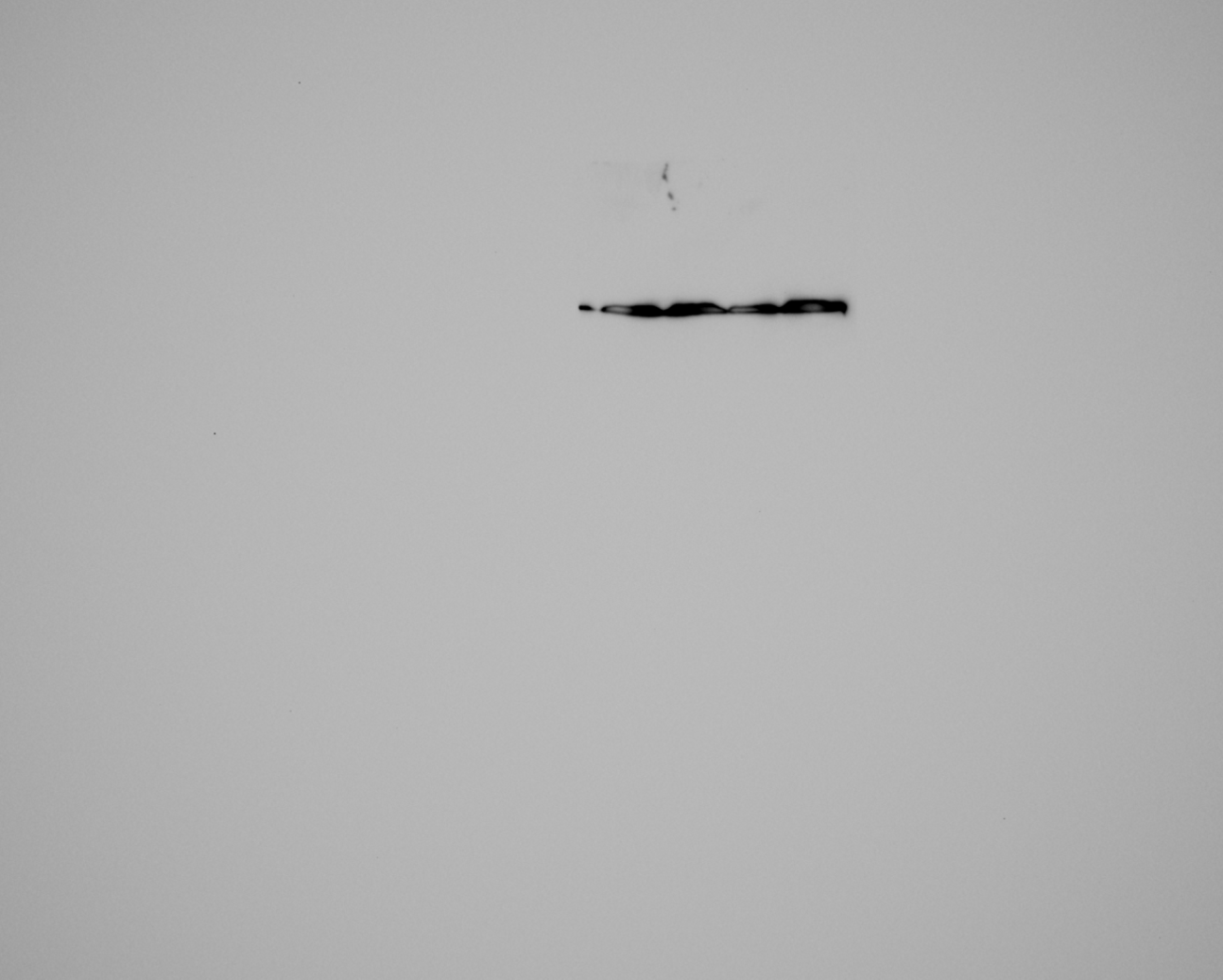
**

**I II III IV**

**I II III IV**

**HPV E7 (SI-S IV)**

**HPV E7 (SI-S IV)**
